# The Administration of Anesthetics in Dental Surgery

**Published:** 1901-01-15

**Authors:** S. Ormond Goldan

**Affiliations:** New York, N. Y.


### THE ADMINISTRATION OF ANESTHETICS IN DENTAL SURGERY.

BY S. ORMOND GOLDAN, M.D., NEW YORK, N. Y.

Read before the New York Odontological Society, May 15, 1900.

From time to time since the discovery of nitrous oxid, ether and chloroform efforts have been made to find a safer anesthetic. It may be questioned whether a greater safety lies in the discovery of newer agents, rather than a more proper knowledge in the use of those at present employed. Operators the world over are realizing more fully every day that it is not so much the anesthetic itself as the administrator in which not only the greatest safety, but superior results lie. Recently there was introduced a mixture of anesthetics, which was used quite extensively in surgery, and to some extent in dentistry. Its so-called great advantages were its safety, rapidity in action, quick return to consciousness and absence of after-effects. Evidently few knew that with ether, properly administered, all the advantages claimed for the new mixture could easily be secured. In fact, more so. This simply depended upon the nature of the operation, the method employed in inducing narcosis, the profundity of and length of time in maintaining it. I might say this mixture of anesthetics is very rarely used at present. I believe the best results in dental as in general surgical work do not lie in the use of any one anesthetic to the exclusion of all others, but in a variation in the anesthetic and methods employed, according to the nature of the operation and peculiarities in the types of patients. While nitrous oxid, ether and chloroform all have their place in dental surgery, nitrous oxid is, and has been, more generally useful, because of its rapidity in producing narcosis and quick return to consciousness. But this also has its disadvantages, in obliging the operator to work rapidly, and so restricts the use of this anesthetic almost exclusively in dental surgery to the extraction of teeth.

The routine use of anesthetics in the longer and painful dental operations has never been attempted, for the reason that the operator can not administer the anesthetic and operate at the same time, except for extractions, where the available working time lasts only from the removal of the face piece to the return of consciousness. To give nitrous oxid in long surgical operations, as compared with oral work, is easy, for here the inhaler may be applied as often as necessary with little discomfort to the surgeon; whereas in dental work it necessitates constant interruptions and loss of time to the operator, which constitutes one of its greatest drawbacks.

Many plans have been advocated for prolonging the anesthesia with nitrous oxid; the most common in dental work in this country is mixing directly with the gas chloroform or ether in variable quantities, the so-called "vitalized air." In my estimation there is no surer method for devitalizing the patient. I consider this mixing of chloroform vapor with nitrous oxid gas doubly dangerous. First, because chloroform is mixed with a deoxygenating agent, and second, it is given in the sitting position, of which I shall have more to say presently. I very seriously question whether anesthesia can be prolonged by this method more than with gas alone, properly used. That the method has been used many times with no mishap does not excuse its use. The most dangerous methods might be employed in many instances with no untoward results. The method should be condemned. Another plan is to give the gas through the nose and mouth. This does prolong the narcosis an appreciable time. Another method is to give the gas very slowly, consuming five or more minutes in the narcosis. By this plan consciousness is not quite so quickly regained. It should be remembered the desire to prolong nitrous oxid anesthesia has been only in connection with tooth extraction. As you no doubt know, the general surgeon will often give an anesthetic to open a simple abscess, or for the removal of sutures. Is it not a remarkable fact that that most painful of all operations, the grinding out of a tooth preparatory to filling, with other painful dental operations, has been, and continues to be done, and the patient obliged to submit to it? There is certainly no more painful operation in surgery. Again, why is so much time consumed in treating a root and preparing the tooth for filling when this, the extraction, if any, the preparation for filling and any other painful oral work could easily be done in one seance, leaving the filling and other unpleasant but not actually painful work to be done at subsequent intervals? Where it takes days and weeks to prepare teeth for filling, it could all be done in a very short time with no interruption to the surgeon, and thoroughly, with the consciousness of having left nothing undone, which no doubt, in spite of every effort, does occur as the result of the inability of the patient to endure the suffering. A good plan, I think, would be to designate by diagram the teeth requiring filling, and also any other painful work; the patient might be anesthetized and all this done at one sitting. I think the time is not far distant when no patient will be expected to submit to this torture, any more than in general surgery work.

Efforts have been made to eliminate the painful procedures in dental practice by the use of local anesthetics. These have not proven successful, and evidently nitrous oxid has not proven itself an entirely satisfactory anesthetic. While we are told of the thousands of times it has been used, (which was principally for extractions) we are not told how often the gas has proven unsatisfactory, and many times failing entirely. While it is well known that nitrous oxid gas and other anesthetic vapors are heavier than air, the practical application of it in dental surgery is, I think, original with myself. This method consists in administering the anesthetic continuously, with no interruptions to the operator. This I have designated, in the absence of a better term, the gravity method. The first two cases anesthetized by me in this manner were, first, the preparation of two teeth for filling; the second, removing a molar and opening the maxillary sinus and removal of some necrosed bone. In both cases gas was administered continuously. There was no cyanosis, stertor or jactitation, and consciousness was immediately regained.

We may consider the use of nitrous oxid alone, or with carbon dioxid, with oxygen, with atmospheric air, with ether and with chloroform. As you are all familiar with the use of nitrous oxid as ordinarily administered, I shall simply mention some salient points in connection with it. While the apparatus can never make the anesthetist, the perfectly working appliances will certainly conduce to the best results. When we examine the rather cumbrous apparatuses generally used in this country for administering nitrous oxid we find little improvement over those of years ago. The stop-cock, for instance, works in two ways, and there is no means of administering ether with gas except to change inhalers. A great defect is the long piece of tubing connecting the gas bag with the face piece, causing an undue stress on respiration. Compare this with the simple, portable, durable and inexpensive device of English manufacture, which leaves little to be desired. Ether can be given without changing inhalers, and, if necessary, it may be arranged for the patient to breathe back and forth into the gas bag. In order to secure best and always satisfactory results, the proper mouth prop should be selected for each case, and so arranged that it may be in position before the inhalation begins. No single mouth prop will answer for all cases. They should be of different sizes and rigid. A suitable lip retractor may be applied, materially helping the operator. When work must be done on each side, the prop should be adjusted to the opposite side before withdrawing the other. To obviate the falling backward of fragments of teeth, etc., into the larynx a small piece of wide mesh gauze should be placed at about the middle of the dorsum of the tongue and separated. This makes, as it were, a hammock to catch all foreign particles. While it is not essential for simple extractions that patients abstain from food for some hours before taking gas, it should be insisted upon where it is intended to administer the anesthetic repeatedly. When more than one application of the mask is necessary, the patient should not be permitted to regain complete consciousness before readmitting the gas. Loose clothing, removal of corsets and all impediments to respiration, absolute quiet, and a third person present always are essentials, I think, well known to all.

*The use of nitrous oxid with carbon dioxid.*—This method can be applied with an apparatus without valves. The one I show was devised by myself; is extremely simple. By this method the patient breathes back and forth into the gas bag. The carbon dioxid is produced during the respiration, and consists in amount equal to that of the oxygen in the blood at the time the inhalation was begun. Where there is not sufficient gas on hand to bring a narcosis to a successful termination, the method may be employed and found useful. It may also be used generally in place of the regular nitrous oxid inhaling apparatus.

The method of using oxygen with nitrous oxid is certainly an improvement over gas alone. This eliminates all the unpleasant features of nitrous oxid. The pulse, respiration and pupil remain approximately normal, and cyanosis is eliminated. The apparatus shown is Dr. Hewitt's, of London. Oxygen is given in quantities varying from about 2 to 12 percent. The combination is particularly useful in delicate types, though often it acts as well in plethoric patients. The method at present is not applicable for the longer narcosis in dental work, because it can not be given continuously. In general surgery, where the inhaler is constantly applied, it acts particularly well, and has been administered by me many times, the longest anesthesia lasting two hours and forty minutes. For simple extraction purposes the narcosis lasts fully as long as with gas alone. Sometimes the available operative period is appreciably prolonged.

We may now consider the use of anesthetics in the longer dental operations, where any work, long or short, may be performed with no interruptions to the operator. This brings us to the use of nitrous oxid, ether and chloroform by our gravity method.

*Nitrous oxid and atmospheric air (diluted oxygen) continuously administered.*—For this purpose the apparatus devised by me is most applicable. The patient should be in the semi or preferably the recumbent posture. Here the inhaler is applied to the nose at about its middle and tilted upward. The gas reaches the patient by its own weight, falls around the nares and into the cup-shaped oral cavity. The patient can be given as much or as little gas as is desirable by letting it flow continuously or not. When air is required, the obturator in the apparatus may be closed and the gas entirely shut off.

It may be argued that the recumbent position is not practicable for the dental operator. I think this objection is more apparent than real. There is really no reason why the dental surgeon can not do his work as well with the patient in the recumbent position as does the general surgeon. When the sitting posture is absolutely essential, or where the method does not act satisfactorily, ether may be employed. There is no serious objection, there being no contra-indication to the employment of ether in the sitting position. The anesthetic is best employed by first anesthetizing the patient with gas, then discontinuing the gas slowly and giving ether gradually, so that by the time the gas has entirely passed off the patient is etherized. The anesthetic is then employed by the gravity method, by giving it from a mask or inhaler applied to the face about the middle of the nose.

The apparatus for giving gas and ether shown is the Barth-Clover, of English make. This appliance is not only the most efficient of the valve apparatuses; it is also the least expensive, even after adding an import duty of 45 percent. The inhaler without valves may be employed, first anesthetizing the patient with gas, then quickly inserting the basket with the gauze saturated with ether. After the patient is etherized the inhaler may be tilted and the anesthetic administered this way, or the mask before mentioned may be employed.

*Chloroform.*—This anesthetic is the last to be used in dental surgery, and should only be employed where there is a condition of the patient contra-indicating gas or ether. Any condition where an increase in arterial tension is undesirable indicates the use of chloroform. These are principally diseases of the circulatory system, as arteritis, atheroma, aneurism, myocarditis, etc.; also in marked renal diseases chloroform acts better. Where gas and ether is not well taken, chloroform may be used. Chloroform should never be directly given to excessively nervous patients. Here gas may first be employed, then ether, to be followed by chloroform. Again, chloroform should never, under any circumstances, be given in any but the recumbent position; nor should the person after anesthetization be changed to the sitting posture, as this may easily lead to syncope. It is a singular fact that the larger proportion of chloroform fatalities occurred before either the patient was entirely anesthetized or the operation begun. And many of these deaths took place in the dentist's chair for simple tooth extraction, the patient being in a sitting position. Chloroform is a vaso-motor paralysant; the recumbent and inverted position tends to a certain extent to overcome a fall in blood pressure. The sudden change from the recumbent to the sitting posture probably accounts for many deaths from this anesthetic. For any dental work which can not be done with the patient in the recumbent posture another agent should be used for anesthesia. Again, no operation, and particularly those of a dental nature, should ever be begun until the patient is thoroughly under the influence of chloroform. With gas and ether the lighter degrees of narcosis very often answer, whereas with chloroform it is particularly essential that all nerve influences be completely abolished to avoid abnormal reflex action. It is said the fifth pair are particularly prone to influence the heart deleteriously and often fatally through vagi, in the lighter stages of anesthesia by chloroform. I think this only partly explains it. There are also, probably, direct reflex influences upon the vaso-motor centers in the medulla. At the same time chloroform causes a direct dilatation of the vessels themselves. After the patient is anesthetized the anesthetic may be administered through the nares by means of a soft rubber catheter, or directly through the mouth by means of a metal tube, or a simple mask may be used and the anesthetic given by the gravity method.

In bringing our discourse to a close we may make a few deductions regarding fatalities. These may be, first, due to improper preparation of the patient, faulty selection and administration of the anesthetic; second, from the anesthetic itself, every precaution having been taken (these are the cases only which should be termed "unavoidable"); third, from some abnormal peculiarity, discoverable or undiscoverable (naturally, if known, it would possibly be avoidable; those should more properly be considered under the first heading); fourth, from hemorrhage, shock, etc., early or late in the narcosis (this differentiates deaths from anesthetics from those while under the influence of them); fifth, coincidence; that is, that the patient would have died at or near the time the anesthetic was administered. Some cases have been reported where death suddenly occurred just before the administration, and these have been termed fright. Coincidence is certainly the rarest of all conceivable contingencies, and I should not mention it at all had I not a most remarkable case to report. This was a patient with marked cardiac disease. A physician in attendance intended to administer chloroform to open a superficial alveolar abscess. When he reached the top of the stairs the nurse suddenly came from the patient's room, telling him the patient suddenly died a moment before. Had he been a few minutes earlier and commenced the administration of the anesthetic I think nearly every one would say the patient died from chloroform. This is certainly an extraordinary occurrence. To those who would explain this as fright I might say the patient was unaware that an anesthetic was to be administered, so had no reason to be frightened. Again, to those who assert that syncope is always due to overdosing, it may be said this patient had an overdose of nothing.—*Dental Cosmos.*

